# Impact of a 6-Week Postpartum Text Messaging Program (Essential Coaching for Every Mother) at 6 Months: Follow-Up Study to a Randomized Controlled Trial

**DOI:** 10.2196/62841

**Published:** 2025-04-02

**Authors:** Justine Dol, Marsha Campbell-Yeo, Megan Aston, Douglas McMillan, Amy K Grant

**Affiliations:** 1 IWK Health, 5850 University Ave, Halifax, NS, B3H 1X1, Canada, 1 9024707706; 2 School of Nursing, Faculty of Health, Dalhousie University, Halifax, NS, Canada; 3 Department of Pediatrics, Faculty of Medicine, Dalhousie University, Halifax, NS, Canada; 4 Maritime SPOR SUPPORT Unit, Halifax, NS, Canada

**Keywords:** mHealth, mobile health, SMS text message, text messages, messaging, self-efficacy, postpartum depression, postpartum anxiety, social support, intervention, postpartum, postnatal, mental health, parenting, mother, depression, anxiety, RCT, randomized controlled trial

## Abstract

**Background:**

Essential Coaching for Every Mother is an SMS text messaging program that positively improved parenting self-efficacy and reduced postpartum anxiety when measured immediately after intervention at 6 weeks postpartum. However, the impact of a short-term postpartum intervention over time is unknown.

**Objective:**

This study aims to compare parenting self-efficacy, postpartum anxiety symptoms, postpartum depression symptoms, and perceived social support at 6 months postpartum for mothers in the Essential Coaching for Every Mother trial.

**Methods:**

Participants (n=150) were randomized to Essential Coaching for Every Mother or control (usual care). Data were collected on parenting self-efficacy (primary outcome, Karitane Parenting Confidence Scale), postpartum anxiety symptoms (Postpartum Specific Anxiety Scale), postpartum depressive symptoms (Edinburgh Postnatal Depression Scale), and perceived social support (Multidimensional Scale of Perceived Social Support) at enrollment and 6-months postpartum. Data were analyzed using analyses of covariance and chi-square analysis.

**Results:**

A total of 139 women completed the primary outcome at 6 months and 136 completed secondary outcomes. At 6 months, there were no statistically significant differences between mothers in the intervention group and mothers in the control group on any of the outcomes. More mothers in the intervention group had higher postpartum anxiety scores (31/68, 45.6%) than mothers in the control group (16/68, 23.5%; *P*=.007).

**Conclusions:**

At 6 months postpartum, all mothers had similar scores on parenting self-efficacy, postpartum anxiety symptoms, postpartum depression symptoms, and social support. Thus, Essential Coaching for Every Mother improved parenting self-efficacy and reduced postpartum anxiety at 6 weeks, with all mothers having similar scores at 6 months postpartum.

## Introduction

### Background

The growth of digital health interventions to address mental health and maternal health outcomes has grown significantly over the past decades [1,2]. Despite this growth, the extent to which preventative digital health interventions improve or maintain outcomes after the intervention has concluded is unclear. This is known as the maintenance effect, which is essential to behavior change interventions and measures the impact of the intervention on outcomes after the treatment has taken place [3]. To date, evidence is mixed as to whether the positive effects of digital health interventions are maintained over time for a variety of health areas including obesity or weight loss [4], smoking [5], and increasing physical activity [6]. However, a meta-analysis on the maintenance effect of SMS text messaging–based interventions found that even after the intervention ends, there is a significant maintenance effect [4]. Furthermore, in the postpartum period, there have been mixed findings as to which type of interventions can maintain behavior change in maternal physical and mental health outcomes [7,8]. Thus, there is a need for reporting follow-up periods of digital health interventions to determine the effectiveness of maintenance postintervention [9] to facilitate the understanding of potential factors that lead to greater maintenance effects and whether ongoing follow-up is needed to maintain effects.

This study reports on the postimplementation outcomes of a 6-week postpartum SMS text messaging program called Essential Coaching for Every Mother*.* This program was designed to send daily SMS text messages to birthing people during the first 6-weeks postpartum who are living in Nova Scotia to improve parenting self-efficacy and perceived social support and to reduce postpartum anxiety and depression symptoms. Essential Coaching for Every Mother was developed through iterative testing with mothers/birthing people (eg, individuals who are physically capable of giving birth) and postpartum health care providers as an evidence-based, SMS text messaging program [10]. In the published results of this randomized controlled trial (RCT), it was found that primiparous women who received the Essential Coaching for Every Mother program had higher parenting self-efficacy at 6 weeks postbirth than those who did not receive the intervention [11]. Additionally, all mothers (regardless of parity) who received the intervention had lower postpartum anxiety symptoms than mothers who did not receive the intervention [11]. This success highlights the effectiveness of the Essential Coaching for Every Mother program to improve immediate parenting self-efficacy and reduce postpartum anxiety symptoms. However, outcomes were measured immediately after completing the intervention at 6 weeks postpartum, and thus, exploration of whether the study effects were maintained is needed to determine whether a short-term intervention has longer-term effectiveness and to determine whether ongoing follow-up is required to maintain positive outcomes.

### Aim

This study aimed to compare parenting self-efficacy, postpartum anxiety symptoms, postpartum depression symptoms, and perceived social support at 6 months postpartum for mothers in the Essential Coaching for Every Mother trial.

### Hypotheses

The following were our hypotheses:

1. Mothers who received Essential Coaching for Every Mother would have higher parenting self-efficacy and lower postpartum anxiety mean scores at 6 months compared with the control group.
2. Mothers who received Essential Coaching for Every Mother would be more likely to have clinically high parenting self-efficacy scores and clinically low postpartum anxiety scores at 6 months, compared with the control group.
3. Given that no significant differences were found in the original trial on postpartum depression symptoms and perceived social support, no differences at 6 months are hypothesized.

## Methods

### Participants

Birthing persons from Nova Scotia, Canada, were recruited remotely through SMS text messages between January 5, 2021, and August 1, 2021. Additional details are available in the study by Dol et al [11].

### Ethical Considerations

This study was approved by the IWK Health Research Ethics Board (#1024984) and Nova Scotia Health Research Ethics Board (#1026534) and is registered with the ClinicalTrials.gov Protocol Registration System (NCT04730570). All participants provided informed consent and were able to opt out at any time.

### Design

This is a follow-up evaluation of a 2-group, stratified, parallel arm RCT which followed a predefined protocol [12]. Participants were recruited from Nova Scotia, Canada via social media advertisements and research study posters at local hospitals and family practice clinics. Participants initiated contact by texting a study phone number to complete a preprogrammed eligibility screening process. All recruitment and onboarding occurred through standardized SMS text messages.

Upon enrollment, participants were first stratified by parity (primiparous and multiparous) and then using a 1:1 allocation, participants were randomized into the intervention or control group. Participants were not blind to their allocation, but hospital staff were. Researchers were aware of allocation but due to the nature of the randomization and remote data collection process, this did not increase any risk of bias.

### Intervention

The Essential Coaching for Every Mother program includes standardized evidence-based SMS text messages that provide information related to newborn care and maternal mental health in the first 6-weeks postpartum [10]. Participants allocated to the intervention are sent messages from birth to 6 weeks postpartum based on the age of their newborn, with 2 messages sent per day in the first 2 weeks (one at 10 AM and one at 5 PM) and a daily message (at 10 AM) for weeks 3 through 6. Participants allocated to the control group did not receive any SMS text messages aside from recruitment and survey requests. No changes to regular care were implemented and no study contact occurred between the 6-week and 6-month surveys.

### Outcome Measures

Participants were invited to complete a survey hosted on Research Electronic Data Capture (REDCap) [13] via SMS text message at enrollment/baseline (preintervention), 6 weeks postpartum (postintervention), and 6 months postpartum (follow-up). For the purposes of this study, only the baseline and 6-month data were used. The primary outcome was parenting self-efficacy measured using the Karitane Parenting Confidence Scale [14]. This 15-item scale assesses the perceived self-efficacy of mothers with newborns from birth to 12 months of age. Scores can range between 0 and 45 and a score of 39 or less is considered to be clinically low perceived parenting self-efficacy [14]. Secondary outcomes included postpartum anxiety symptoms (Postpartum Specific Anxiety Scale [PSAS] [15]), postpartum depression symptoms (Edinburgh Postnatal Depression Scale [EPDS] [16]), and perceived social support (Multidimensional Scale of Perceived Social Support [17]). For the PSAS, the clinical cut-off for postpartum anxiety symptoms is 112 out of a possible 201 [15] and for the EPDS, a score of 9 or above indicates depressive symptoms in a community sample and 13 or greater indicates probable clinical depression [16,18]. Therefore, higher scores in both the PSAS and EPDS indicate higher symptomology of postpartum anxiety and depression, respectively. No clinical cut-off scores are available for the Multidimensional Scale of Perceived Social Support.

### Data Analysis

Data were analyzed on a per-protocol analysis, excluding women who requested to stop receiving the messages or did not return the 6-month follow-up survey. A series of analyses of covariances were conducted to examine the effects of the intervention on the outcomes of interest at 6 months postpartum considering allocation. In all analyses of covariances, parity, maternal age, and scores on the respective outcomes at baseline were entered as co-variates (ie, when analyzing parenting self-efficacy, parity, maternal age, baseline parenting self-efficacy scores were included as co-variates).

Chi-square analysis was conducted to compare whether participants scored above or below the clinical cut-off that identifies low parenting self-efficacy or high postpartum anxiety and depression symptoms. For the chi-square analysis, all participants who completed the outcome survey at 6 months were included. A *P* value of .05 was considered significant for all outcomes. SPSS (version 29.0; IBM SPSS Statistics) was used for analysis.

## Results

### Overview

**Figure 1.**
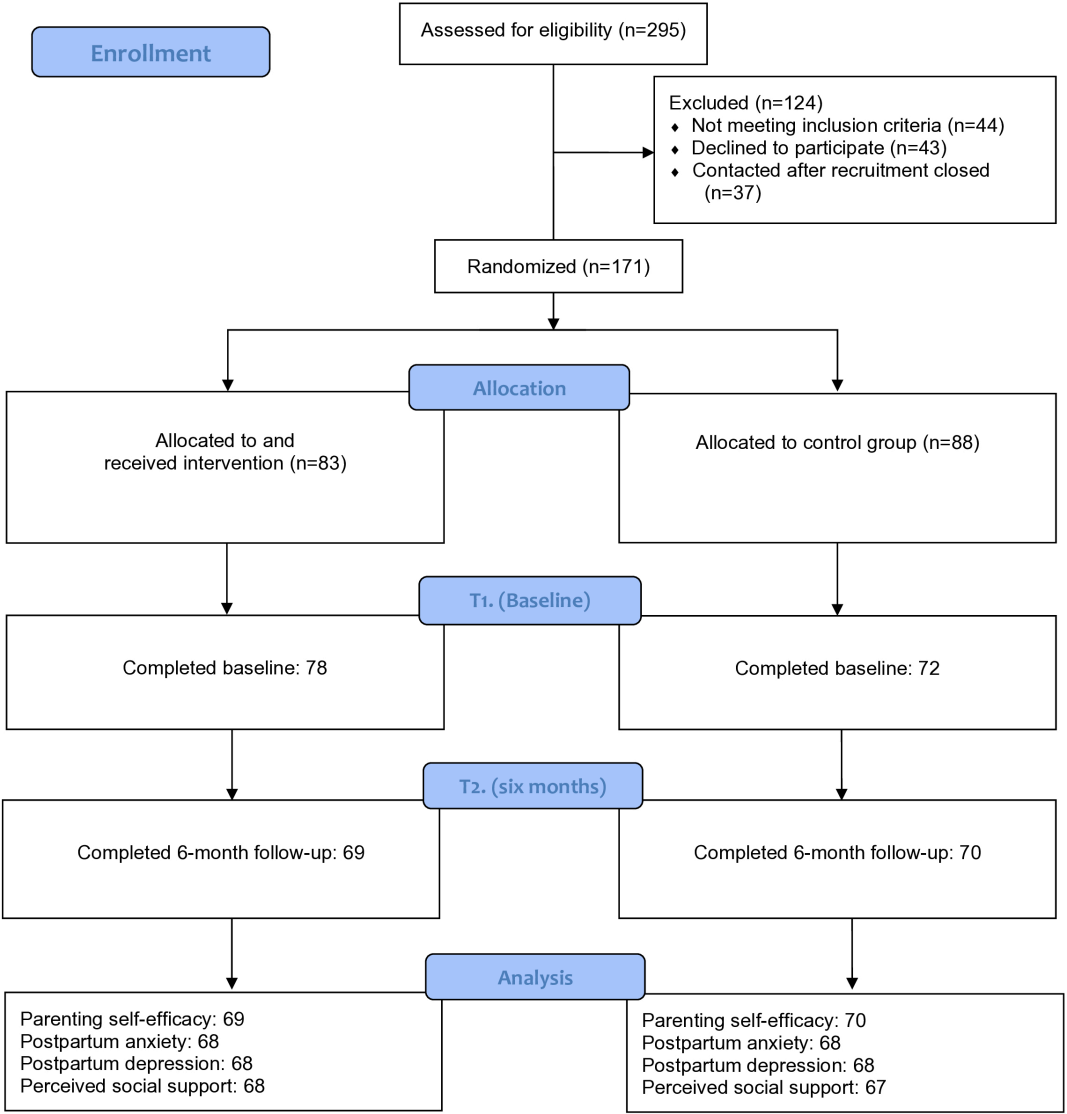
Consolidated Standards of Reporting Trails (CONSORT) flowchart.

Of the 171 participants randomized, 150 participants completed the baseline survey and were enrolled in the study. Of those enrolled, 139 (81.2%) participants completed the 6-month follow-up survey and were included in this analysis (Figure 1). All participants identified as cis-gendered females and as mothers; thus, the term “mother” will be used in describing the sample. Mothers were predominantly married, White, and had an undergraduate degree or higher. At 6 months, the groups were similar in all demographics, except for race, with mothers in the control group being more likely to identify as White compared with mothers in the intervention group. This differed from baseline, as there was no difference in race but mothers in the control group were significantly older than mothers in the intervention group (*P*=.053) [11]. At baseline, there were no differences between the groups on any of the primary outcomes [11]. Additional demographic details are available in Table 1 and Dol et al [11].

**Table 1.** Baseline characteristics for the intervention and control groups.

Demographics	Intervention (n=69)	Control (n=70)	*P* value
Maternal age (years), mean (SD)	30.7 (4.8)	32.1 (4.1)	.07^a^
History of depression or anxiety, n (%)	24 (34.8)	18 (25.7)	.27^b^
Marital status, n (%)			.71^b^
Single or not living with partner	3 (4.3)	4 (5.7)	
Married, common-law, or living with partner	66 (95.7)	66 (94.3)	
Household income in CAD^c^, n (%)			.06^b^
Less than 74,999 (US $ 52,286)	26 (37.7)	16 (22.9)	
75,000-149,999 (US $52,287-US $104,573)	31 (44.9)	34 (48.6)	
Over 150,000 (US $104,574)	8 (11.6)	17 (24.3)	
Prefer not to answer	4 (7.2)	3 (4.3)	
Education n (%)			.31^b^
High school or incomplete college or university	4 (5.8)	9 (12.9)	
College diploma	16 (23.2)	10 (14.3)	
Undergraduate degree (BA, BSc)	31 (44.9)	35 (50.0)	
Graduate degree (MSc, PhD)	17 (24.6)	16 (22.9)	
Prefer not to answer	1 (1.5)	—^d^	
Race, n (%)			.02^b^
White	58 (84.1)	67 (95.7)	
Non-White (included Black, Chinese, Filipino, Latin American, Greek, and Indigenous)	11 (15.9)	3 (4.3)	

a Conducted using independent *t* test.

b conducted using Pearson chi-square analysis.

c Conversion CAD to USD estimated at 1 CAD=US $0.70.

d Not applicable.

### Parenting Self-Efficacy

Based on the 139 mothers who completed parenting self-efficacy measures at 6 months, there was no statistically significant difference in parenting self-efficacy at 6 months postpartum based on allocation (*F*_1,134_=0.112, *P*=.74, partial η^2^=0.001) (Table 2). A chi-square analysis was conducted to determine whether the proportion of mothers who had high (≥40) or low parenting self-efficacy scores (≤39) at 6 months were different based on group allocation. There were no differences in the proportion of mothers with high or low parenting self-efficacy scores based on allocation at 6 months postpartum (*χ*^2^_1_=0.091, *P*=.76) (Table 3).

**Table 2.** Adjusted means scores based on allocation at 6 months postpartum.

Outcome	Intervention, mean (SD)	Control, mean (SD)	*P* value
Parenting self-efficacy (n=139)	40.14 (3.73)	40.11 (3.94)	.74
Postpartum anxiety scores (n=136)	106.71 (20.97)	99.35 (19.13)	.30
Postpartum depression scores (n=136)	10.01 (4.66)	8.19 (4.94)	.20
Social support (n=135)	5.82 (0.95)	5.82 (1.12)	.79

**Table 3.** Comparison of high and low scores at 6 months postpartum by allocation.

Outcome	Intervention, n (%)	Control, n (%)	*P* value
Parenting self-efficacy^a^			.76
High (40 or above)	47 (68.1)	46 (65.7)	
Low (39 or below)	22 (31.9)	24 (34.3)	
Postpartum anxiety^b^			.007
High (112 or above)	31 (45.6)	16 (23.5)	
Low (111 or below)	37 (54.4)	52 (76.5)	
Postpartum depression^b^			.12
High (9 or above)	42 (61.8)	33 (48.5)	
Low (8 or below)	26 (38.2)	35 (51.5)	
Postpartum depression^b^			.08
High (13 or above)	17 (25)	9 (13.2)	
Low (12 or below)	51 (75)	59 (86.8)	

a Intervention (n=69) and control (n=70).

b Intervention (n=68) and control (n=68).

### Postpartum Anxiety Symptoms

Based on 136 mothers who completed the postpartum anxiety measure at 6 months postpartum, there was no statistically significant difference in postpartum anxiety scores at 6 months postpartum (*F*_1,131_=1.077, *P*=.30, partial η^2^=0.00) (Table 2). These findings differed in the chi-square analysis, where mothers in the intervention group were more likely to have clinically high (≥112) postpartum anxiety scores (31/68, 45.6%) compared with those in the control group (16/68, 23.5%) (*χ*^2^_1_=7.315, *P*=.007) (Table 3).

### Postpartum Depression Symptoms

Based on the 136 mothers who completed the postpartum depression measure at 6 months, there was no statistically significant difference in postpartum depression mean scores at 6 months postpartum based on allocation (*F*_1,131_=1.680, *P*=.20, partial η^2^=0.013) (Table 2). In the chi-square analysis comparing mothers with scores ≥9 to ≤8, there were no differences in the proportion of mothers with high or low postpartum depression symptoms at 6 months postpartum (*χ*^2^_1_=2.408, *P*=.12). Additionally, comparing participants who scored ≥13 or ≤12, there were no differences in the proportion of mothers with high or low postpartum depression symptoms (*χ*^2^_1_=3.043, *P*=.08).

### Perceived Social Support

Based on the 135 mothers who completed the perceived social support measure, there was no statistically significant difference in perceived social support scores at 6 months postpartum based on allocation (*F*_1,130_=0.071, *P*=.79, partial η^2^=0.001) (Table 2).

## Discussion

### Principal Results

This study sought to explore mothers’ parenting self-efficacy, postpartum anxiety symptoms, postpartum depression symptoms, and perceived social support at 6 months postpartum, after receiving Essential Coaching for Every Mother, a 6-week postpartum SMS text messaging intervention immediately after birth. At 6 months, all mothers, regardless of their allocation, had similar scores on all outcomes. Mothers in the intervention group were slightly more likely to have high postpartum anxiety symptoms compared with mothers in the control group. The implications of these findings are discussed below.

For the primary outcome of parenting self-efficacy, the hypotheses were not supported in the analysis. The hypotheses were that mothers who received Essential Coaching for Every Mother would have higher mean parenting self-efficacy scores compared with mothers in the control group and would be more likely to have parenting self-efficacy scores that would be considered high (≥40). While there were no significant differences in scores or differences in high scores, both the intervention and control groups had mean scores that would be considered “high” parenting self-efficacy. In the RCT measuring immediate intervention effectiveness [11], primiparous women who received the Essential Coaching for Every Mother program had a greater increase in parenting self-efficacy than those who did not receive the program. This may suggest that Essential Coaching for Every Mother was able to improve parenting self-efficacy during the earlier postpartum period, particularly for primiparous women, showing potential to improve immediate parenting self-efficacy during an early critical period. Given the relatively high parenting self-efficacy scores at 6 months postpartum, it is clear that parenting self-efficacy increases over time in the first 6 months as mothers become more comfortable and confident in their parenting role [19]. Early interventions for primiparous mothers may be helpful in bridging the gap to achieve earlier parenting self-efficacy.

In relation to postpartum anxiety symptoms, the original RCT found that postpartum anxiety symptoms decreased at 6 weeks for women who received the Essential Coaching for Every Mother program, regardless of parity, compared with the control group [11]. Thus, the hypothesis was that mothers who received Essential Coaching for Every Mother would have lower mean postpartum anxiety scores at 6 months as well as more mothers would have clinically low postpartum anxiety scores compared with the control group. Like parenting self-efficacy, there were no significant differences at 6 months between the groups on average postpartum anxiety scores. However, mothers in the intervention group were more likely to have a clinically high postpartum anxiety score compared with mothers in the control group. Postpartum anxiety symptoms have been found to be higher in mothers with an infant 4‐6 months than mothers with an infant 0‐3 months [20], suggesting that perhaps there may be factors that influence a later emergence of anxiety symptoms during the postpartum period. Additionally, it has been suggested that postpartum anxiety may have a u-shaped relationship, with mothers who have higher anxiety levels during pregnancy experiencing a dip when their infant is born and then continue to increase back up to pregnancy levels up to 24 months postpartum whereas mothers with low anxiety levels during pregnancy tend to stay low across the postpartum period [21]. In this study, anxiety scores at baseline were higher for the intervention group, so there may be an influence of self-referral bias whereby mothers who were more anxious were interested in enrolling in the study. Particularly since the COVID-19 pandemic, the loss of support and ability to consult with health care providers or friends has also been associated with higher levels of mental health concerns [22]. Combined with normal fluctuation that occurs in the postpartum period along with decreased social support and potentially being predisposed to higher anxiety, there may be factors other than the Essential Coaching for Every Mother program that resulted in increased anxiety symptoms for intervention mothers. Despite the higher levels in the Essential Coaching for Every Mother group, both groups had a mean below the clinical cut-off for high levels of postpartum anxiety. At the individual level, it is important to ensure that birthing people have access to ongoing mental health support throughout the postpartum period.

Last, no differences in postpartum depression symptoms and perceived social support at 6 months were hypothesized since there were no significant differences found in the original trial at 6 weeks postpartum. This was found in both analyses, with no differences in postpartum depression symptoms and social support at 6 months. Despite the lack of significant findings, mothers in the intervention group did have higher postpartum depression mean scores, and more mothers were in the >9 group than mothers in the control group. During the recruitment for the RCT, participants were not excluded if they were currently experiencing mental health concerns or had a history of mental health concerns. While randomization is expected to equalize this between groups by design, this may not have been sufficient to balance this across groups as the intervention group had higher, yet not significant, postpartum depression symptoms at baseline as well [11]. Additionally, the intervention group had a higher, although again not significant, difference in having a history of mental health concerns (24/69, 34.8% vs 18/70, 25.7%) which may have influenced their postpartum depression symptoms at 6 months postpartum.

### Limitations

This follow-up analysis is limited by the small sample size and loss of follow-up, which may have impacted the ability to be sufficiently powered in the analyses. While the original study was sufficiently powered, this follow-up study lost some power as participants who did not complete the 6-month timepoint were removed. This study was carried out in Canada in English, and findings may be different in other populations. We were unable to explore other variables that influenced mothers’ psychosocial and mental health scores across the postpartum period. Mothers who participated in the study may not be fully representative of the population, as they were predominately White, highly educated, and high-income earners. Additionally, our sample may have been influenced by self-referral bias, as participants were remotely recruited and signed themselves up for participation and there was a greater number of participants in the control group who dropped out before completing the baseline questionnaire [11]. Given these limitations, the findings should be interpreted in this light.

### Comparison With Prior Work

Questions remain about the appropriate dose and engagement of postpartum SMS text messaging interventions to improve psychosocial and mental health outcomes. The first year after an infant is born is associated with significant changes in physical and emotional outcomes for mothers. Risk factors for postpartum depression and anxiety are wide-ranging and include, but are not limited to, having depression during pregnancy [23-25], having a history of depression [23,26], or experiencing abuse or marital conflict [24,25]. Additionally, evidence shows that postpartum anxiety varies across the first 6 months of the postpartum period, ranging from 14.8% to 17.8% [27]. In addition, well established is the comorbidity between anxiety, depression, social support, and parenting self-efficacy in the postpartum period [19,28], suggesting that postpartum adjustment is multifaceted and interdependent. More research is needed to understand whether ongoing support throughout the postpartum period may be able to alter mental health outcomes as well as other interventions that may address additional risk factors. Given that Essential Coaching for Every Mother was designed primarily to improve parenting self-efficacy, it is important that other supports are available to mothers that target mental health outcomes beyond the initial 6-weeks.

There is a challenge with designing interventions that improve mental health outcomes in the postpartum period, particularly in regard to universal support to women considered low risk [29]. In a recent scoping review analyzing 70 unique evidence-based universal interventions to support parents between conception and 12 months postpartum, only half reported evidence of effectiveness against their reported outcome measures, suggesting a need for a multifaceted approach to support parent well-being across the perinatal period [30]. Another review examining digital health interventions for postpartum anxiety and depression found that digital health interventions significantly reduced postpartum depression and postpartum anxiety symptoms [31]. It is also important to consider what might make an intervention effective beyond the treatment period. In analyzing the maintenance effectiveness of physical activity and dietary interventions for adults, Fjeldsoe et al [32] found that intervention characteristics that were associated with maintenance effects were those of longer duration (>24 weeks), face-to-face contact, the use of a variety of intervention strategies, and the use of follow-up prompts. Examining parent training more broadly, providing booster sessions or intermittent contact postintervention, have been suggested as potentially effective strategies to maintain intervention effects over time [33].

While digital health interventions are desired and accepted by postpartum mothers [34], interventions designed for implementation during the postpartum period should take into consideration how interventions can maintain positive impacts over time. In a meta-synthesis of what women want in the postpartum period, Finlayson et al [35] clearly summarize: “To cope with this period of adjustment women express the need for practical, emotional and psychological support from family members, peer groups and online sources, as well as from health providers. Women also want information and reassurance from health providers delivered in a consistent manner by authentic, familiar providers who recognise the mother’s as well as baby’s needs, within a well-resourced and flexible healthcare system that respects their cultural context*”*. Given the multiple challenges that emerge for mothers during the postpartum period, both in-person and digital support from reliable sources of information that respond to mothers’ needs and infant development is needed. Engaging patients in the development of perinatal digital health solutions is important to improve health outcomes but is not yet common practice [36-38].

### Future Directions

Future work should explore the potential to expand the timeline of the Essential Coaching for Every Mother program beyond the immediate 6-week period, as there are clearly challenges parents experience beyond this time frame that are impacting their psychosocial and mental health. Additionally, future work should consider the population that may be in most need of such an intervention, such as primiparous mothers or those with higher postpartum anxiety and depression scores at baseline. Future work should identify at-risk groups and determine whether any differences between low-risk and high-risk groups can be improved with the Essential Coaching for Every Mother program.

### Conclusions

In conclusion, mothers, regardless of whether they received Essential Coaching for Every Mother, a 6-week postpartum intervention, had similar scores on parenting self-efficacy, postpartum anxiety symptoms, postpartum depression symptoms, and social support at 6 months. This suggests that the Essential Coaching for Every Mother program was able to improve parenting self-efficacy for primiparous mothers and reduce postpartum anxiety symptoms in the immediate postpartum period. At 6 months, both groups were similar, indicating that support during the immediate 6-week postpartum period is critical to ensure early intervention.

## Supplementary material

10.2196/62841 Checklist 1 CONSORT-eHEALTH checklist.

## Abbreviations

EPDS: Edinburgh Postnatal Depression Scale
PSAS: Postpartum Specific Anxiety Scale
RCT: randomized controlled trial
REDCap: Research Electronic Data Capture
